# A new species of *Psathyrella* (Psathyrellaceae, Agaricales) from Italy

**DOI:** 10.3897/mycokeys.52.31415

**Published:** 2019-05-16

**Authors:** Giovanni Sicoli, Nicodemo G. Passalacqua, Antonio B. De Giuseppe, Anna Maria Palermo, Giuseppe Pellegrino

**Affiliations:** 1 Department of Biology, Ecology and Earth Science The University of Calabria Cosenza Italy; 2 Museum of Natural History of Calabria and Botanical Garden, The University of Calabria, 87036 Arcavàcata di Rende, Cosenza, Italy The University of Calabria Cosenza Italy

**Keywords:** Agaricomycetes, Basidiomycota, Fen-sedge, Marshes, southern Italy, Taxonomy

## Abstract

Sporophores of a new *Psathyrella* species have been reported for the first time as growing at the base of *Cladiummariscus* culms in the Botanical Garden of the University of Calabria, Rende, Cosenza, southern Italy. The fungus was initially identified as *P.thujina* (= *P.almerensis*) by means of both ecology and macro- and microscopic characteristics of the basidiomes, then referred to *P.cladii-marisci* sp. nov. after extraction, amplification, purification and analysis of the rDNA ITS region. We came to this conclusion after comparing our specimen with the descriptions of the taxa available in the literature for the genus *Psathyrella*.

## Introduction

Within the cosmopolitan fungal genus *Psathyrella* (Fr.) Quél. (*Agaricales*, *Psathyrellaceae*), about one hundred species have traditionally been recognised in Europe, almost all saprotrophs and found in many and diverse environments. Either terrestrial or lignicolous, they grow mainly on organic debris from various origins, such as dung, post-fire locations and dead stems of larger herbaceous plants (Vesterholt and Knudsen 1992). *Psathyrella* basidiomes are pileate, stipitated and exannulate or, at most, with a fugacious ring and the hymenophore is gilled, pale pink when young, turning brown with age due to a dark spore print. Moreover, they have, as the etymology indicates, a very fragile and ephemeral consistency. Despite these common macroscopic characters of the basidiomes, a recent phylogenetic analysis revealed the extremely complex origin of this genus, recognising species as belonging to a *Psathyrellasensu stricto* group or to *P. sensu lato* complex, the former including 19 clades and the latter involving eight genera (*Coprinellus*, *Kauffmania*, *Cystoagaricus*, *Typhrasa*, *Lacrymaria*, *Homophron*, *Coprinopsis*, *Parasola*), thus consistently widening the list of such “psathyrelloid” basidiomycetes (Örstadius et al. 2015).

During an investigation on the mycoflora of the Botanical Garden at the University of Calabria (Rende, Cosenza, Italy), basidiomes of an apparently “psathyrelloid” fungus were detected at the base of a fen-sedge [*Cladiummariscus* (L.) Pohl (*Cyperaceae*)], a cosmopolitan-distributed plant species (Lansdown et al. 2018) occurring in marshy places of most Italian regions (Bartolucci et al. 2018), but rarely in southern Italy.

Based on records reported by Örstadius et al. (2015), nine clades of *Psathyrellas.s.* include species associated with moist soils and marshy places: “*spadiceogrisea*” (four species), “*fibrillosa*”, “*noli-tangere*” and “*prona*” (two species each), “*candolleana*”, “*cystopsathyra*”, “*lutensis*”, “*obtusata*” and “*pygmaea*” (one species each). Nevertheless, only three species have been found to be growing on sticks or on remnants of hygrophilous plants: *P.lutensis* (Romagn.) Bon, as a monospecific “*lutensis*” clade, *P.thujina* A.H. Sm. (=*P.almerensis* Kits van Wav.) in the “*spadiceogrisea*” clade and *P.typhae* (Kalchbr.) A. Pearson & Dennis in the “*candolleana*” clade.

The aim of this work was therefore to identify our basidiomes by using both morpho-ecological and biomolecular tools. This was highly encouraged by the habitat peculiarity and the close relationship with a plant species with which no species of *Psathyrellaceae* had ever been found associated.

## Materials and methods

**Figure 1.**
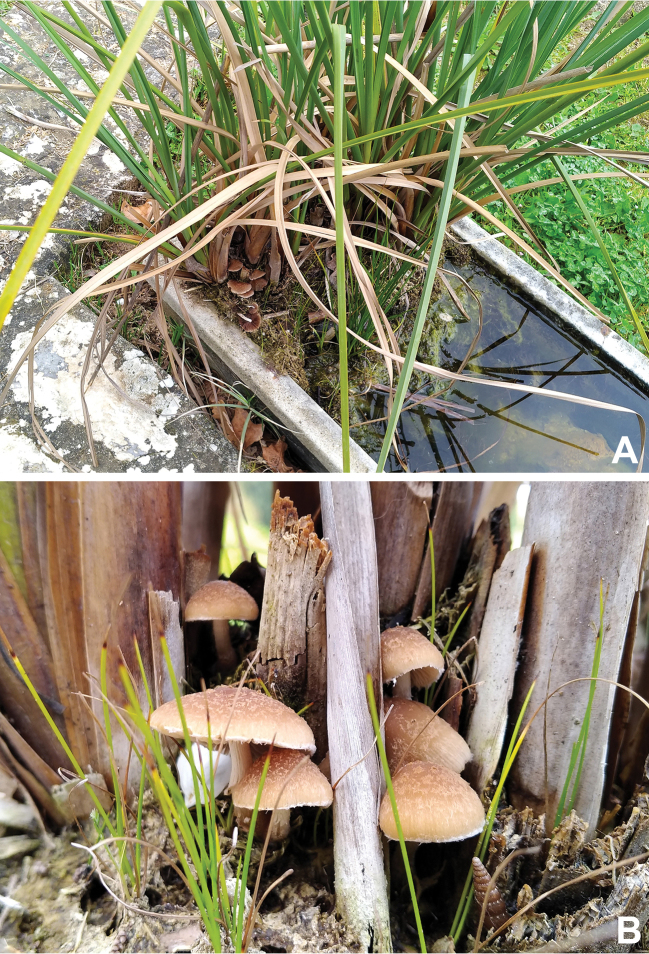
A tuft of *Cladiummariscus* planted in a tank at the Botanical Garden of the University of Calabria, southern Italy (**A**), and first-sight features of *Psathyrella* basidiomes at the base and in-between of remnants of excised culms of the plant (**B**).

Eight basidiomes of the above “psathyrelloid” fungus were observed and collected on 10 April 2018, as gregarious all around and at the base of *Cladiummariscus* cut culms (Fig. 1). In 2012, that plant had been removed, together with the whole clump of mud attached to its roots, from a natural marsh named Lago dell’Aquila (Laureana di Borrello, Reggio Calabria, southern Italy) and transplanted to the Botanical Garden at the corner of a 90 × 37 cm-wide and 30 cm-deep concrete tank, which had permanently been kept full to the brim with water. Since then, some leaves of water lily (*Nymphaeaalba* L.) have been introduced to float on the water surface inside the tank and the mud mass has been increasing, while the *C.mariscus* plant has been expanding and producing new culms that are cut every year.

### Morphology

The basidiomes were first macroscopically examined for features, colours, sizes, hymenophore shape, pileus and stipe ornamentations, smell and taste. Then, the structures of the basidiome were microscopically inspected for cheilo- and pleurocystidia occurrence and features, presence of clamp connections, basidia and spore features. These observations were carried out under a light microscope (Axioplan 2 Imaging Microscope, Carl Zeiss, Germany) at 400 and 1,000 magnifications on fragments of *pileipellis* and gills placed on slides in 10% NH_4_OH. The results were compared with those published in the morphological keys for the *Psathyrella* species and, more specifically, with those species reported as the closest, according to morphology and ecological site conditions, i.e. *P.thujina*, *P.typhae* and *P.lutensis* (Kits van Waveren 1985, Vesterholt and Knudsen 1992, Christan et al. 2017, Henrici 2017).

### DNA Extraction, Amplification and Sequencing

One of the basidiomes was dehydrated at room temperature and destroyed for molecular analysis: DNA extraction, amplification, purification and sequencing of the nuc rDNA internal transcribed spacer region (ITS). DNA extraction was implemented by using CTAB protocol (Doyle and Doyle 1987) and the ITS region was amplified using the primer combination ITS1F/ITS4 (White et al. 1990). The polymerase chain reaction (PCR) was performed in a 25-µl reaction volume containing 1.0 µl DNA, 2.5 µl 10 × 5-Prime–MasterMix Buffer (Thermo Fischer Scientific, Waltham, Massachusetts, USA) and 1.25 µl of each primer (10 µM/µl). The PCR was carried out according to the following amplification programme: 3 min initial denaturation at 94 °C, 35 cycles (30 s denaturation at 94 °C, 1 min annealing at 55 °C, 45 s extension at 72 °C) and a 10 min final extension at 72 °C. This programme was carried out in a T1000 Thermocycler (Biometra, Goettingen, Germany). The PCR products were purified using a QIAquick PCR purification kit (Qiagen Inc., Valencia, California, USA). Sequencing was performed by means of a Bigdye terminator cycle sequencing kit (Applied Biosystems, Foster City, California, USA). The sequencing reaction was run by BMR Genomics (Padua, Italy) on a 96-capillaries ABI 3730XL DNA Sequencer.

Forward and reverse DNA fragment electropherograms were checked by means of the CHROMAS 2.6.5 software (technelysium.com.au) for a complete reconstruction of the ITS1, ITS2 and 5.8 gene fragments. Ambiguous regions at the start and the end of the alignment were deleted and gaps were manually adjusted to optimise the alignment. The sequence generated for this study is deposited in GenBank with the code MK080112.

### Alignment and Phylogenetic Analysis

Consensus sequences were generated from both forward and reverse primer reads in the BioEdit sequence alignment editor, version 7.2.5 (Hall 1999), then homology searches were performed at the National Centre for Biotechnology Information (NCBI) Web site using BLAST. This sequence was then compared with those of the *Psathyrella* species deposited in GenBank on which the phylogenetic analysis had recently been performed (Padamsee et al. 2008, Battistin et al. 2014, Örstadius et al. 2015, Yan and Bau 2018). A total of 45 ITS sequences, including three *Coprinellus* spp. (Table 2) were aligned using MAFFT with the L-INS-i option (Katoh et al. 2017). The aligned ITS dataset consisted of 702 nucleotide sites (including gaps). FASTA alignments from MAFFT were loaded in IQ-TREE 1.5.6 (Nguyen et al. 2014) to perform Maximum Likelihood Analysis. Clade robustness was assessed using a bootstrap (BT) analysis with 1,000 replicates (Felsenstein 1985). Phylogenetic trees were visualised using the FigTree v1.3.1 (Rambaut 2009).

## Results

### Morphology

**Figure 2.**
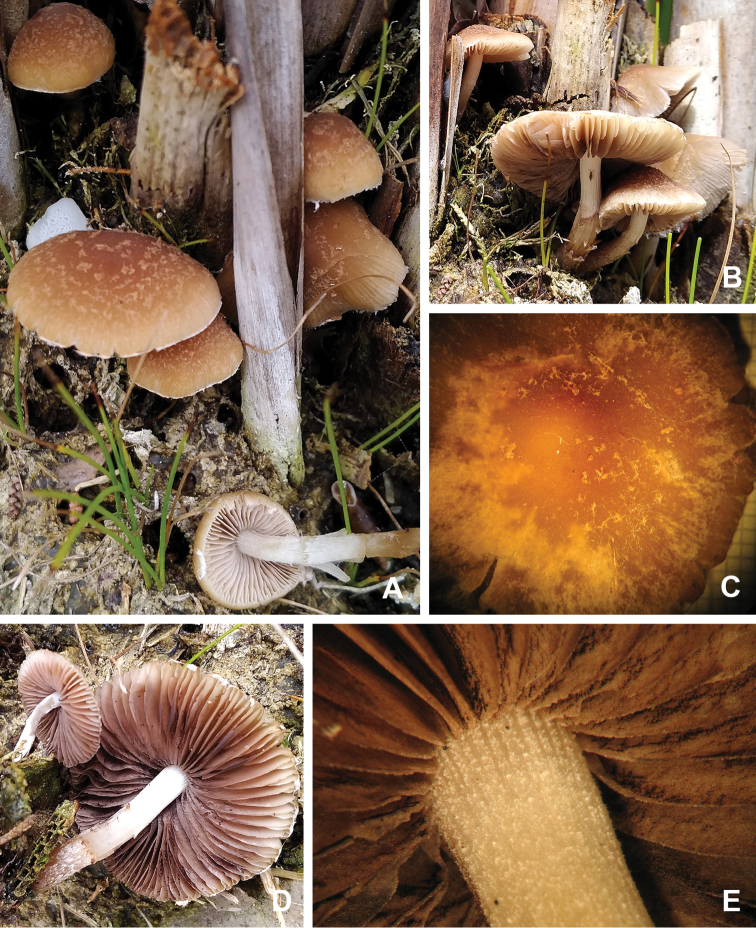
Macro-morphological characteristics of the *Psathyrella* basidiomes: scales of velar origin on pilei tops and margins, and beige-coloured gills (**A**); cylindrical, white and exannulate stems under a lateral profile (**B**); colour-shading of a cap hygrophany and fibrillose details of velar-originated scales (**C**); gills turning brown-purplish with spore maturation and a fibrillose surface of a stem base (**D**); a pruinose stem apex bearing a mature hymenophore with white gill edge lines (**E**).

**Figure 3.**
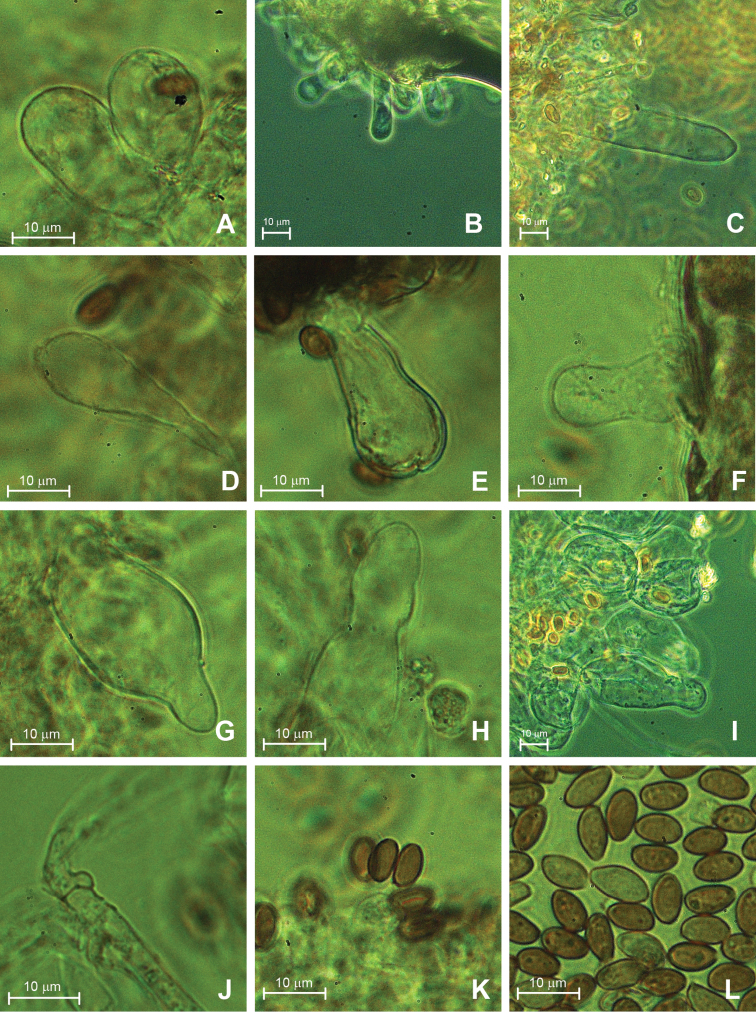
Micro-morphological characteristics of the *Psathyrella* mycelium: clavate and sphaeropedunculate (**A**), and cylindric (**B, C**) cells at a gill edge; differently clavate (**D, E**) and utriform (**F**) cheilocystidia; variously utriform-shaped pleurocystidia (**G, H, I**); a fibulate hypha (**J**); a 4-spored basidium (**K**); basidiospores (**L**).

The macro- and micro-morphological features of the basidiomes collected at the base of the fen-sedge plant in the Botanical Garden are shown in Figures 2, 3. At first sight, by observing the macro-level characters, i.e. the small-medium size, the extreme fragility at handling and the brown-blackish spore print, the basidiomes were easily assigned to the *Psathyrella* genus (Vesterholt and Knudsen 1992). Secondly, the occurrence of sphaeropedunculate and clavate cells along the gill edge and the utriform shape of some cheilo and pleurocystidia seemed to direct them to the Section SpadiceogriseaeKits van Wav.,subsectionSpadiceogriseae (Romagn.) ex Kits van Wav. (Kits van Waveren 1985).

If we compare the morphological features of our specimens with those belonging to the closest *Psathyrella* species, a number of differences emerge (Table 1). Our specimens appeared to be more similar to *P.thujina* (Henrici, 2017), previously described as *P.almerensis* (Kits van Waveren 1985, Vesterholt and Knudsen 1992), except for the pileus diameter reaching 3.5 cm in our specimens, but never exceeding 2.5 cm in this species. Furthermore, our *Psathyrella* revealed versiform-shaped cheilocystidia, while those reported for *P.thujina* are only utriform. *P.typhae* was also divergent for the pileus diameter, not exceeding 2.5 cm, but even for pileus and stipe colours and for lacking pleurocystidia. On the other hand, the mucoid deposits, characterising the pleurocystidioid cheilocystidia of *P.lutensis*, were absent in our specimens. In addition, the spore length range was wider in our specimens than in *P.thujina* and *P.lutensis* and all the closest three species, which showed larger spores on average.

**Table 1.** Main differences between our *Psathyrella* sp. and the closest species, according to the morphological characteristics of basidiomes and mycelium, and ecology. (Differences from our specimen are in bold characters).

**Morpho-ecological characteristics**	***Psathyrella* sp.**	*** P. thujina ***	*** P. typhae ***	*** P. lutensis ***
Pileus diameter (cm)	3.5	**2.5**	**2.5**	**4.0**
Pileus colour	Hazelnut brown, then beige brown	Warm brown, then beige brown	**Pinkish-ochre brown, then pale flesh brown**	**Dark reddish brown, then very pale brown**
Stem colour	White with a pruinose apex	White with a pruinose apex	**Whitish to pale brown**	**White with a pruinose apex, brownish base**
Spore size (µm)	7.2–11.8 x 4.3–6.0	**9.0–11.5 x 4.5–6.5**	**7.5–11.5(12.0) x 5.5–8.0**	**9.0–10.0 x 4.5–5.5**
Cheilocystidia	Versiform, chiefly utriform	**Utriform**	Versiform, chiefly utriform	Versiform, chiefly utriform
Pleurocystidia	Utriform	Utriform	**Absent**	**Versiform, chiefly utriform to ventricose**
Mucoid deposits on cystidia	NO	NO	NO	**YES**
Habitat	Marshes, on cut culms of *Cladium*	Marshes, on cut culms of *Typha*, *Phragmites*, *Cirsium*, *Epilobium*	Marshes, on cut culms of *Typha*, *Epilobium*, *Scirpus*, *Phragmites*, *Rumex*, *Iris*	**Deciduous forests, on sticks in mud**
Seasonal occurrence	Spring	**Autumn to winter**	**Summe**r	**Summer to autumn**

As for ecology, the plant genus *Cladium* Browne has never been reported as a substrate to any other *Psathyrella*, although *P.thujina* and *P.typhae* are commonly found on the remnants of ecologically similar plants (Kits van Waveren 1985, Vesterholt and Knudsen 1992, Örstadius et al. 2015, Henrici 2017). Furthermore, the genus *Cladium* was not mentioned in the unique Italian report of *P.thujina*, which was found “in open sites, close to any hygrophilous plants” (Voto 2016), in accordance with Henrici (2017) who refers this species to reed-beds and generic damp marshy habitats. Finally, our specimen was collected in the spring, whereas the above three other *Psathyrella* species seem to occur in other seasons.

### DNA Analysis

**Figure 4.**
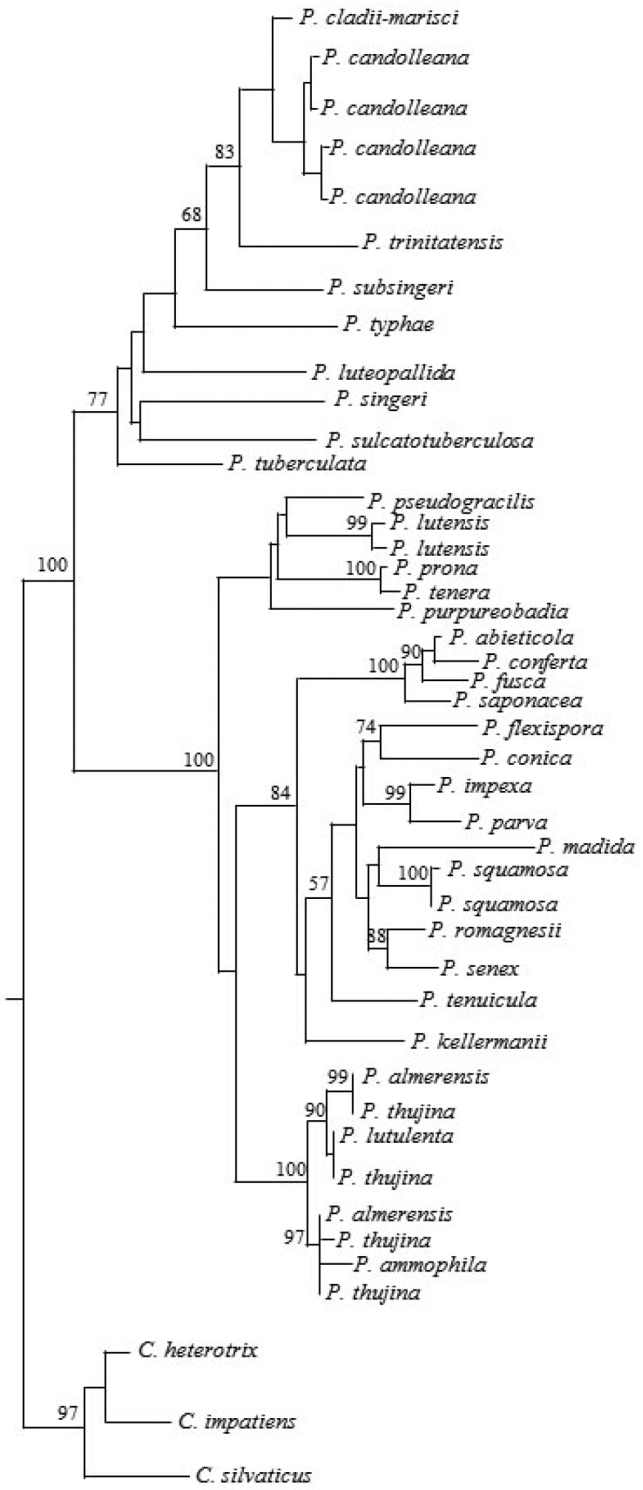
One of the most parsimonius trees from the phylogenetic analysis of *Psathyrella* spp. based on nrDNA sequence data. Bootstrap values are shown above branches based on 1,000 replicates (values below 50 are not shown).

The obtained nrDNA sequence was 702 bp long. By comparing it with those published in GenBank, we obtained a data matrix composed of 44 taxa and 710 characters, 276 gap-free sites and 240 conserved sites. The highest homology (99%) was observed with *P.candolleana* (Fr.) Maire, which was confirmed by the phylogenetic analysis (Fig. 4). Indeed, the phylogenetic tree shows that our specimen falls into the “*candolleana*” clade, such a heterogeneous group, including taxa from different morphology, ecology and geographic provenance and, amongst them, the above-cited *P.typhae* (Battistin et al. 2014, Örstadius et al. 2015, Yan and Bau 2018).

**Table 2.** Species used for the phylogenetic analyses including GenBank Accession Numbers and published references.

**Species**	**GenBank accession No.**	**Reference**
* Psathyrella abieticola *	KC992891	Örstadius et al. 2015
* P. almerensis *	KC992874	Örstadius et al. 2015
* P. almerensis *	KC992873	Örstadius et al. 2015
* P. ammophila *	KC992872	Örstadius et al. 2015
* P. candolleana *	AB306311	Ogura-Tsujita and Yukawa 2008
* P. candolleana *	DQ389720	Larsson and Örstadius 2008
* P. candolleana *	MG734719	Yan and Bau 2018
* P. candolleana *	MG734720	Yan and Bau 2018
* P. cladii-marisci *	MK080112	This study
* P. conferta *	KC992890	Örstadius et al. 2015
* P. conica *	MG734713	Yan and Bau 2018
* P. flexispora *	MF966494	Heykoop and Moreno 2002
* P. fusca *	MF966503	Heykoop and Moreno 2002
* P. impexa *	KC992900	Örstadius et al. 2015
* P. kellermanii *	KC992920	Örstadius et al. 2015
* P. luteopallida *	MG734736	Yan and Bau 2018
* P. lutensis *	MG734748	Yan and Bau 2018
* P. lutensis *	DQ389685	Larsson and Örstadius 2008
* P. lutulenta *	KC992875	Örstadius et al. 2015
* P. madida *	KC992932	Örstadius et al. 2015
* P. parva *	KC992912	Örstadius et al. 2015
* P. prona *	KJ939634	Larsson and Örstadius 2008
* P. pseudogracilis *	KC992853	Örstadius et al. 2015
* P. purpureobadia *	NR_119670	Larsson and Örstadius 2008
* P. romagnesii *	DQ389716	Larsson and Örstadius 2008
* P. saponacea *	MH155965	Yan and Bau 2018
* P. senex *	MG734732	Yan and Bau 2018
* P. singeri *	MG734718	Yan and Bau 2018
* P. squamosa *	KC992939	Örstadius et al. 2015
* P. squamosa *	MG367206	Yan and Bau 2018
* P. subsingeri *	MG734714	Yan and Bau 2018
* P. sulcatotuberculosa *	KJ138423	Battistin et al. 2014
* P. tenera *	FJ899635	Frank et al. 2010
* P. tenuicula *	DQ389706	Larsson and Örstadius 2008
* P. thujina *	KC992873	Örstadius et al. 2015
* P. thujina *	KC992874	Örstadius et al. 2015
* P. thujina *	KY680791	Örstadius et al. 2015
* P. thujina *	KY680792	Örstadius et al. 2015
* P. trinitatensis *	KC992882	Örstadius et al. 2015
* P. tuberculata *	MH497604	Yan and Bau 2018
* P. typhae *	DQ389721	Larsson and Örstadius 2008
* Coprinellus heterothrix *	FM878018	Nagy et al. 2011
* C. impatiens *	FM163177	Nagy et al. 2011
* C. silvaticus *	KC992943	Örstadius et al. 2015

## Discussion and conclusions

Based on results from both morphological and molecular analysis, our collection cannot be assigned to a known species. According to morphology, our *Psathyrella* should be closer to *P.thujina* (Section Spadiceogriseae). By contrast, the DNA ITS sequence would undoubtedly include it in the “*candolleana*” clade, where each species showed up to a 99% ITS sequence similarity with our sample. The most widespread and known species in this clade, *P.candolleana* and *P.leucotephra* (Berk. & Broome) P.D. Orton, both commonly occurring in Europe, too, are however morphologically very different from our specimen, by forming large pilei (diameter up to 8.0 cm) and lacking pleurocystidia; furthermore, the latter frequently even shows a torn annulus in the upper part of the stem, which we did not observe in our *Psathyrella* (Kits van Waveren 1985, Vesterholt and Knudsen 1992, Consiglio 2005). The “*candolleana*” clade encompasses two more European species according to two recent phylogenetic analyses (Nagy et al. 2011, Battistin et al. 2014): *P.sulcatotuberculosa* (J. Favre) Einhell., previously regarded as a variety of *P.typhae* (Kits van Waveren 1985), which mainly differs from our *Psathyrella* and from *P.typhae* itself with a partially-sulcate and -tuberculate pileus surface, and *P.badiophylla* (Romagn.) Bon which forms spores normally exceeding 10–11 µm in length (Kits van Waveren 1985, Vesterholt and Knudsen 1992); in addition, both also lack pleurocystidia, which was considered to be such a morphologically relevant character to induce the establishment of the Section Spintrigerae within the subgenus Psathyra (Fr.) Sing. ex Kits van Wav. (Kits van Waveren 1985). Moreover, except for *P.typhae*, which is the only *Psathyrella* ecologically comparable to our collection, all the above species are reported to grow in diverse site conditions, i.e. close to stumps of trees or on branches, on moist ground, in grass, on mossy woods or on various other vegetable matter (Kits van Waveren 1985, Vesterholt and Knudsen 1992). Finally, as far as we know, other species in the “*candolleana*” clade are even geographically more distant, each colonising a different kind of organic debris (Padamsee et al. 2008, Örstadius et al. 2015, Yan and Bau, 2018).

Therefore, within this framework, the placement of our fungus into the “*candolleana*” clade, together with other species showing strong differences for geographic and ecologic reasons, should not prevent the recognition of a new *Psathyrella* species.

Anyhow, more and more scientific contributions are remarking that the genetic analysis of a fungus aiming at taxonomic purposes can alone generate artefacts, i.e. “false positive” or “chimeras”, especially when such analysis is implemented by using a unique gene (Thines et al. 2018, Lücking et al. 2018). A polyphasic approach, i.e. based on the combination and integration of all the available informative data (Colwell 1970), is becoming more and more desirable for taxonomic research in mycology, whereas the ITS rDNA region is still considered as the universal genetic marker for fungi (Schoch et al. 2012).

On the basis of the outcomes deriving from the morphologic, ecologic and biomolecular characteristics which we have identified in this note, we are therefore inclined to establish a new species of *Psathyrella*.

## Taxonomy

### Psathyrella cladii-marisci

Taxon classificationFungiORDOFAMILIA

Sicoli, NG Passal., De Giuseppe, Palermo & Pellegrino sp. nov.

Figs 1 , 2 , 3

#### Etymology.

The specific epithet derives from *Cladiummariscus*, the name of the plant where it was first detected.

#### Diagnosis.

Similar to *P.thujina* from which it differs by showing a larger pileus (about 40% larger), a wider range of spore length, versiform cheilocystidia and basidiomes occurring in spring.

#### Holotype.

Italy. Calabria, Cosenza, Rende, Orto Botanico Università della Calabria. 39°21'25.05"N, 16°13'44.57"E, 220 m a.s.l., marsh at the base of cut culms of a *Cladiummariscus* (L.) Pohl plant, transplanted from Lago dell’Aquila (Laureana di Borrello, Reggio Calabria, southern Italy) at the corner of a concrete tank maintained full of water, 10 April 2018, Antonio Biagio De Giuseppe & Giovanni Sicoli (CLU F302).

#### Description.

*Habit* psathyrelloid. *Pileus* up to 3.5 cm diam., conical-convex when young, hemispheric to applanate at maturity, with a deeply striate margin, hazelnut in colour, turning to pale beige when dry. *Pileipellis* with evident concentric arachnoid fibrils of velar origin, whitish and easily removable, often exceeding the cuticle margin. *Lamellae* distant, ventricose, adnate, intermingled with numerous lamellulae, initially pale pink, then intensely brown-purplish. *Lamella edge* whitish with numerous sphaeropedunculate cells. *Stipe*, very fragile, cylindrical, white, exannulate with a diffuse fibrillosity especially on the basal surface, apical surface pruinose. *Basidiospores* 7.2–11.8 × 4.3–6.0 µm (n = 100), ellipsoid to ovoid-ellipsoid, with a thick and smooth wall, adaxially flattened with a central 2µm-wide germ pore and a distinct hilar appendix. *Spore-print* dark brown. *Basidia* clavate, 4-spored. *Cheilocystidia* versiform, often utriform, seldom cylindrical to clavate. *Pleurocystidia* utriform-shaped. *Mycelium* septate and clamped. *Context* with apparently no smell, taste mild.

#### Habit, habitat and distribution.

In small groups (gregarious), on the culm remnants of *Cladiummariscus*. So far, known only from the type locality.

## Conclusions

This probably rare and, apparently, never before detected species could occur more commonly if further surveys confirmed a sort of preference for *C.mariscus* as a growing substrate for the fungus. This plant was observed all over Italy (Bartolucci et al. 2018), although becoming more and more scattered due to the progressive surface reduction of its natural growing environment, i.e. marshes and wet sites quite close to the sea at mid-low altitudes. These sites have been long subjected to draining and other forms of anthropogenic land uses. Since human activities have been causing a deep influence and restriction on density and distribution of the spontaneous flora, including *C.mariscus*, the gradual depletion of plant biodiversity in such sites could also result in negative effects on fungal diversity, thus rendering even more scarce the occurrence of basidiomes of such taxa as *P.cladii-marisci* in Italy.

## Supplementary Material

XML Treatment for Psathyrella cladii-marisci
