# Corrigendum: Sicoli G, Passalacqua NG, De Giuseppe AB, Palermo AM, Pellegrino G (2019) A new species of *Psathyrella* (Psathyrellaceae, Agaricales) from Italy. MycoKeys 52: 89–102. https://doi.org/10.3897/mycokeys.52.31415

**DOI:** 10.3897/mycokeys.58.38856

**Published:** 2019-10-02

**Authors:** Giovanni Sicoli, Nicodemo G. Passalacqua, Antonio B. De Giuseppe, Anna Maria Palermo, Giuseppe Pellegrino

**Affiliations:** 1 Department of Biology, Ecology and Earth Science, The University of Calabria, 87036 Arcavàcata di Rende, Cosenza, Italy The University of Calabria Cosenza Italy; 2 Museum of Natural History of Calabria and Botanical Garden, The University of Calabria, 87036 Arcavàcata di Rende, Cosenza, Italy The University of Calabria Cosenza Italy

## Abstract

Not required

Two morphological features of the new *Psathyrella* species, described in the paper, need to be emended: the presence of pleurocystidia and the spore length range.

Figure 3 (G, H and I) showed pyriform cells on the cap cuticle, not pleurocystidia. This mistake originated from a wrong labelling of the photo folder. Conversely to what is stated in Table 1 and on pages 93 and 100, pleurocystidia were not observed. Coherently with the DNA analysis, this correction addresses the discussion on page 99 to the certain inclusion of our fungus in the Section *Spintrigerae* (Fr.) Konr. & Maubl. and in the “*candolleana*” clade.

Due to a transcription error, the spore length range was 7.2–8.8 µm instead of 7.2–11.8 µm.

Missing reference (cited in the Introduction as Lansdown et al. 2018):

Lansdown RV, Juffe Bignoli D, Beentje HJ (2017) *Cladium mariscus*. The IUCN Red List of Threatened Species 2017: e.T164157A65910896. https://www.iucnredlist.org/species/164157/65910896

